# Identity of Microfilariae Circulating in Dogs from Western and South-Western Romania in the Last Decade

**DOI:** 10.3390/pathogens10111400

**Published:** 2021-10-29

**Authors:** Simona Giubega, Mirela Imre, Marius Stelian Ilie, Kálmán Imre, Iasmina Luca, Tiana Florea, Gheorghe Dărăbuș, Sorin Morariu

**Affiliations:** 1 Department of Parasitology and Dermatology, Banat’s University of Agricultural Sciences and Veterinary Medicine “King Michael I of Romania” from Timisoara, Calea Aradului 119, 300645 Timisoara, Romania; simonagiubega@gmail.com (S.G.); mirela.imre@gmail.com (M.I.); iasminaluca0@gmail.com (I.L.); suici.tijana@gmail.com (T.F.); gheorghe.darabus@fmvt.ro (G.D.); sorin.morariu@fmvt.ro (S.M.); 2 Department of Animal Production and Veterinary Public Health, Banat’s University of Agricultural Sciences and Veterinary Medicine “King Michael I of Romania” from Timisoara, Calea Aradului 119, 300645 Timisoara, Romania; kalmanimre@usab-tm.ro

**Keywords:** *Dirofilaria*, *Acanthocheilonema*, dogs, dirofilariosis, Romania

## Abstract

*Dirofilaria* infections in dogs are recognized as widespread mosquito-borne diseases with zoonotic potential, that are caused by the filarioid nematode (*Onchocercidae*) species *Dirofilaria immitis* and *Dirofilaria repens*. The long-term survey conducted in western and south-western Romania was undertaken in order to provide valuable data on the occurrence of *Dirofilaria* infections in dogs. Blood samples from 1088 dogs, originating from 73 localities of 11 western and south-western counties in Romania, were collected and examined using the modified Knott test. Subsequently, all of the microscopically positive samples were subjected to molecular analysis for confirmation. Altogether, the data obtained showed the percentage of dogs with circulating microfilariae to be 21.42% (233/1088) of dogs tested in the investigated region. The identified species, in cases of monoinfections, were *D. immitis*, *D. repens*, and *Acanthocheilonema reconditum* in 106 (9.74%) samples, 102 (9.38%) samples, and 1 (0.09%) sample, respectively. Twenty-four (2.21%) samples were simultaneously positive for *D. immitis* and *D. repens*. There was no association (*p* > 0.05) between infection status and breed; however, sex and lifestyle were positively associated (*p* < 0.05) with the percentage of dogs with circulating microfilariae and might be regarded as risk factors for infection. The results of the present investigation indicate potential zoonotic risks for humans living in the screened area and support the imperative to increase awareness among both veterinarians and physicians, regarding the continuous spread of these zoonotic filariae.

## 1. Introduction

*Dirofilaria* infections in dogs are widespread mosquito-borne diseases caused by filarioid nematodes (*Onchocercidae*) with a zoonotic potential—namely, *Dirofilaria immitis* and *Dirofilaria repens* [1,2].

*Dirofilaria immitis* and *Dirofilaria repens* are two of the most well-known vector-borne helminths, both exerting a severe influence on veterinary care and public health, due to the emergence of canine cardiopulmonary and subcutaneous dirofilariosis in both dogs and humans [3].

While some *Dirofilaria*-infected dogs do not show any clinical signs of disease, others may exhibit clinical signs of variable intensity, depending on the implicated filarial species. *Dirofilaria immitis*, the etiological entity causing canine heartworm disease (HD) is commonly found in pulmonary arteries. Worms can also be found in the right cardiac ventricle, the right atrium, and (in worst case scenarios/in advanced stages of the disease) in the vena cava. The disease has a severe impact on veterinary medicine due to its clinical signs which include, among others (depending on the evolution), coughing, dyspnoea, weakness, and death. *Dirofilaria repens*, an agent that generally causes subclinical infections, is responsible for subcutaneous dirofilariosis in dogs and it is frequently linked with the presence of adults in subcutaneous tissues, representing the main cause of human dirofilariosis. The clinical signs of this infection in dogs include pruritus, localized alopecia, or cutaneous ulceration [3,4,5,6,7,8,9]. 

The diagnosis of canine heartworm disease is based on the corroboration of results from detailed clinical examinations with a combination of different diagnostic methods, including revealing the presence of blood microfilariae, radiographic examinations, echocardiography, rapid antigen tests, ELISA, or polymerase chain reaction (PCR) [10,11,12].

The detection and identification of circulating microfilariae, the morphological and molecular identification of adult parasites, and the cytological and histological analysis of nodules can all be used to make a *D. repens* diagnosis [7].

Canine dirofilariosis is an endemic disease in many European countries [13], including Romania, where the prevalence of *D. immitis* has been reported to range between 0% and 60%, depending on the surveyed area and the diagnostic tool used [14,15,16,17,18,19]. Recent studies performed on Romanian territory offer information regarding the presence of this parasite in other regions found at considerable distances from the areas included in the present study. The results of our study aim to complete the epidemiological map of the *Dirofilaria*-circulating microfilariae that are spread among the canine population. The goal of our study was to provide updated information regarding the occurrence of *Dirofilaria*-circulating microfilariae in dogs from western and south-western Romania within the last decade, as well as data regarding the risk factors involved in the acquisition of *Dirofilaria* infections in Romania.

## 2. Results

The screening of the 1088 canine blood samples from the western and south-western areas of Romania—aiming to identify the *Dirofilaria* species—revealed an overall frequency rate of 21.42% (233/1088) for canine dirofilariosis (detailed in Table 1). 

The identified species in case of monoinfections were *D. immitis*, *D. repens* and *Acanthocheilonema reconditum* in 106 (9.74%) samples, 102 (9.38%) samples, and 1 (0.09%) sample, respectively. Twenty-four (2.21%) samples exhibited the simultaneous presence of *D. immitis* and *D. repens*.

**Figure 1:**
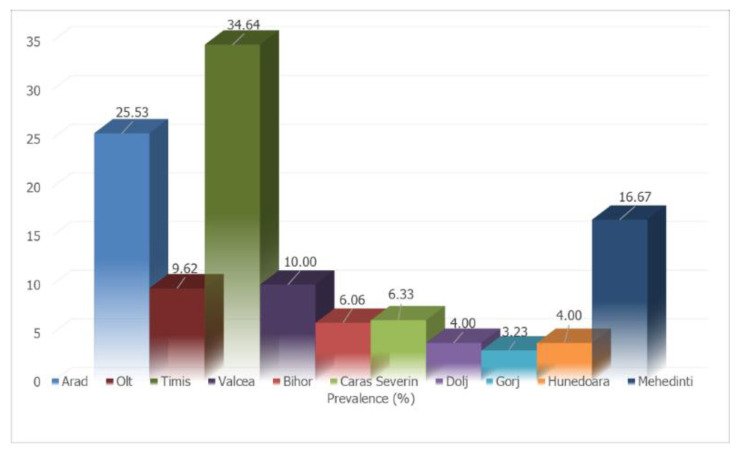
Percentage distribution of dogs positive for *Dirofilaria*-circulating microfilariae in western and south-western Romania.

**Figure 2:**
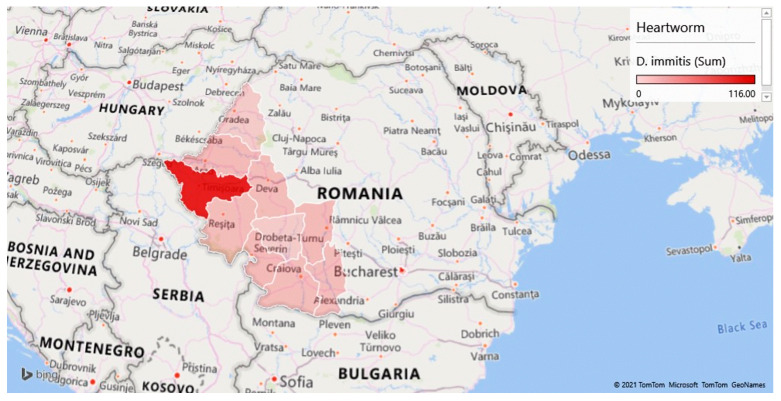
Geographical distribution of dogs positive for *Dirofilaria immitis* in western and south-western Romania.

**Figure 3:**
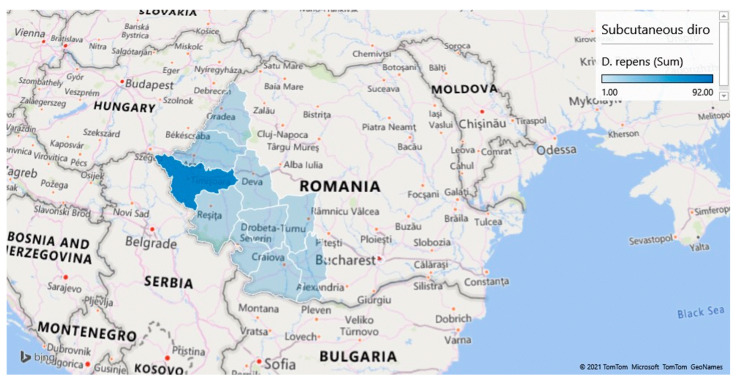
Geographical distribution of dogs testing positive for *Dirofilaria repens* in western and south-western Romania.

The screening of samples coming from the various counties included in the study led to the identification of prevalence rates ranging from 3.23% (2/62) in Gorj to 34.64% (186/537) in Timiș (Table 1 and Figure 1, Figure 2 and Figure 3). 

**Figure 4:**
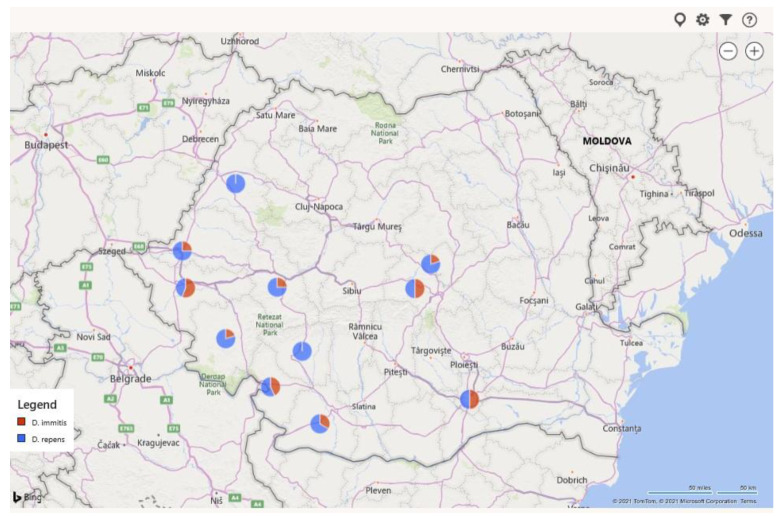
Geographical distribution of canine *Dirofilaria*-circulating microfilariae in western and south-western Romania.

**Figure 5:**
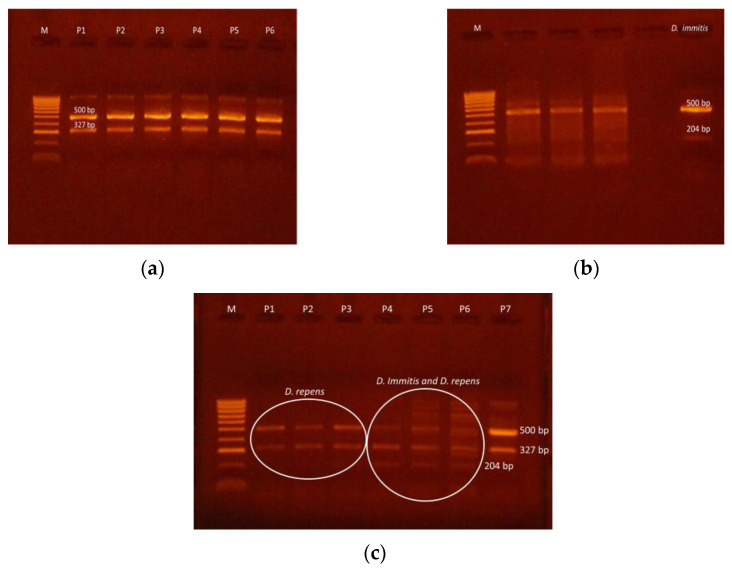
Migration bands of products resulted from multiplex–PCR during molecular diagnostic: (**a**) *Dirofilaria repens* (M—molecular weight–size marker; P1–6—positive samples); (**b**) *Dirofilaria immitis* (final column) M—molecular weight–size marker); (**c**) *Dirofilaria repens* and *Dirofilaria immitis*.

*Dirofilaria repens* was identified in 126 (11.58%) samples following PCR examination (Figure 4). The assay was based on the amplification of a region of the 12S rDNA gene. The 1.5% agarose gel migration products of the PCR multiplex highlighted bands of consistent width, with obvious bright bands at 500 bp (genus diagnostic) and 327 bp (Figure 5a,c). In contrast, the other 130 samples (11.95%) generated migration bands characteristic for the *D. immitis* species at 500 and 204 pair bases (Figure 5b,c). 

The comparative statistical analysis of the positive and negative cases revealed several age-related aspects (Table 2 and Table 3). The risk of contracting *Dirofilaria* infections appears to increase with age, while animals below 1 year of age are not considered at risk. 

The statistical difference between positive and negative cases was highly significant in terms of sex distribution. This applied to urban–rural distribution of cases as well as to the categories of dogs with owners and dogs from shelters. 

There were no statistically significant differences between the positive and negative animals in terms of breed distribution. 

## 3. Discussion

Several published studies and case reports mention the infection with *Dirofilaria* in Romania, revealing various prevalence rates among dogs [14,15,16,17,18,19], cats [20,21], or wild carnivores [22] from different areas. The present study, conducted under extensive spatial (almost a quarter of all counties (11/41)) and temporal conditions (over a 10 year period) brings updated and valuable information on this subject.

In Europe, infection with *Dirofilaria* spp. in dogs was identified in many countries including Austria [12], the Baltic countries [23], Bulgaria [24], the Czech Republic and Slovakia [25], France [26], Greece [6,27], Hungary [28], Italy [29,30,31], Serbia [32], and Spain [33], with minor differences among reports [13]. An up-to-date and accurate picture of *Dirofilaria* infections in central and northern European countries is provided by a study by Fuehrer et al., 2021, which states that, although the number of studies has increased in recent years, epizootiological knowledge is fragmented [34].

Outside Europe, dirofilariosis was detected in Thailand [35,36], French Guyana [37], Nepal [38], Iran [39], and the USA [8,40]. 

Anvari et al. (2020) [41] evaluated the global status of *D. immitis* infections in dogs, based on data available in literature (8.78% in Romania, 10.45% in Europe, and 10.91% across the world). Genchi and Kramer (2020) [13] reported the prevalence rates of *Dirofilaria immitis* and *D. repens* in the Old World (between 3.6% and 42% in Romania).

Information regarding prophylactic therapy was not available for all studied dogs. It is widely acknowledged that the use of macrocyclic lactones leads to the disappearance of microfilaria from the blood stream, a fact which could influence the estimation process of the real prevalence rates. The use of immunochromatography or ELISA can compensate this underestimation though highlighting circulating antigens released by gravid *Dirofilaria* females. Underestimation may also occur if the animals are parasitized with only males, immature females, or if there are less than two gravid females which fail to produce detectable levels of antigens. The possibility for cross-reactions should also be taken into account, as well as the presence of antigen–antibody complexes that can be hidden following therapy based on macrocyclic lactones and can end up influencing the results of quick tests or ELISA assays. The modified Knott method is considered the gold standard diagnostic of *Dirofilaria* infections; moreover, there is an excellent correlation rate between this method, ELISA, and PCR tests [42,43,44,45,46]. 

On the other hand, animals infected with various helminths, such as *Dipetalonema reconditum*, *Dirofilaria repens*, *Ancylostoma caninum*, and *Trichuris* spp., have shown potential cross-reactions. Furthermore, it has been suggested that the antigens of *A. vasorum* and *D. immitis* may share epitopes, which might lead to cross-reactions in antigen detection assays. Consequently, for epidemiological investigations in dogs with a suspected heart worm infection, the use of highly specific diagnostic techniques at the same time is suggested [47].

Statistical analysis showed that the risk factors were represented by age (above 1 year of age), sex, habitat, and whether the animals had an owner or came from the shelter. Breed and age factors (less than 1 year of age), were not considered risk factors.

When it comes to age as a risk factor in infections with *Dirofilaria,* there are several studies that support this aspect [15,48,49,50,51,52] and studies that did not reach any significant conclusions [53].

Regarding sex, males were considerably more affected (statistically) than females, a fact that is supported by Traversa et al., 2010 [54], but is contradicted by Ciucă et al., 2016 [15] and Muñoz et al., 2020 [53].

The origin of the animals (either urban or rural) and whether they had an owner or they came from the shelter have significantly influenced the prevalence of the infection with *Dirofilaria*. It can be considered that the animals from the rural environment are more exposed to the attack of vectors over longer periods of time, compared to animals from the urban environment. However, we cannot overlook the fact that owners from the urban environment tend to be more careful when it comes to their pets, compared to owners from the rural environment. The fact that animals from shelters do not benefit as much from deworming treatments, and the fact that dirofilariasis prevention has been faulty prior to their arrival in the shelter, has significantly influenced the possibility of infection with *Dirofilaria* spp.

A limitation in the molecular or parasitological diagnosis of canine heartworm disease is directly related to the fact that both methods target microfilariae. Occult infections with *D. immitis* can represent about 20–30% of all cases. In this situation, it is recommended to perform a method of detection/identification of microfilariae (Knott) as well as an antigen test [6,10,37,43,55,56,57].

In our study, we can discuss the issue of underestimation regarding the prevalence of *D. immitis*, an aspect that cannot be discussed for *D. repens*.

## 4. Materials and Methods

**Figure 6:**
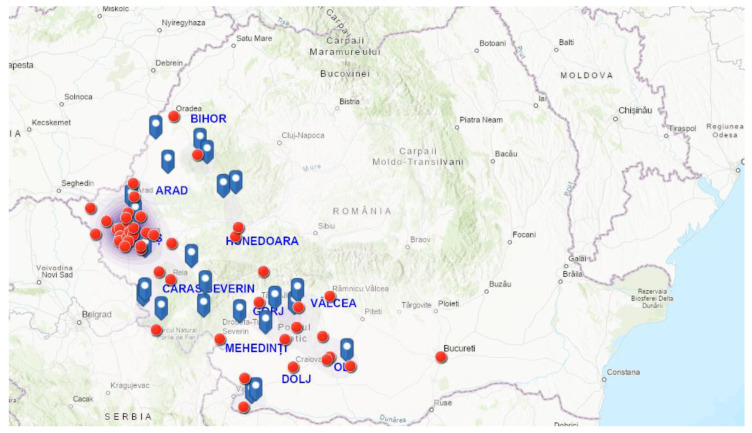
Map of Romania showing locations of sampling, where *Dirofilaria* positive (red dots) and negative (blue dots) dogs were identified.

The survey was conducted on a total of 1088 dogs from 11 counties in western and south-western Romania. The screened animals came from 73 localities (Figure 6) distributed at a countywide level as follows: Arad (5 localities), Bihor (5 localities), Timiș (27 localities), Caraș-Severin (9 localities), Hunedoara (5 localities), Mehedinți (2 localities), Dolj (4 localities), Gorj (5 localities), Olt (4 localities), Vâlcea (6 localities), and Bucharest (southern area of Romania), respectively. 

The dogs included in the study, were randomly selected and they either came from shelters (60 dogs—5.5%), were patients brought in for consultation at the University Veterinary Clinics of the Faculty of Veterinary Medicine from Timisoara (728 dogs—66.9%) or from they were patients in private veterinary practices (300 dogs—27.6%). The dogs were brought in for their annual vaccines, routine check-ups, or when presenting with various clinical signs. 

The owners of dogs that were registered at the University Veterinary Clinics have signed an informed consent for the use of data, while the owners from private practices expressed their consent verbally.

A blood sample was collected from each animal, in properly labelled 3 mL vacutainer tubes containing anticoagulant (EDTA), and all blood samples were sent to the parasitology laboratory from the University Veterinary Clinics for testing and species identification.

The modified Knott’s technique [45] was performed on all samples in order to highlight the presence of microfilariae and for species differentiation. Subsequently, positive samples were confirmed through polymerase chain reaction (PCR), according to instructions by Gioia et al., 2010 [58].

The DNA was isolated from the blood samples using the PureLink™ Genomic DNA mini kit (Invitrogen™, Carlsbad, CA, USA), according to the manufacturer’s instructions. Following extraction, the DNA samples were deposited at −20 °C until further processing. Subsequently, the samples were subjected to the polymerase chain reaction procedure (PCR).

Thus, the basic technique consisted of the use of the PCR multiplex, based on an equimolar combination between a pair of general nematode primers (500 bp)—12SF (5-GTTCCAGAATAATCGGCT A-3) and 12SRdeg (5-ATTGACGGA TG(AG)TTTGTACC-3)—and a pair of specific primers for *Dirofilaria immitis* (204 bp) and *Dirofilaria repens* (327bp)—12SF2B (5-TTTTTACTTTTTTGGTAATG-3) and 12SR2 (5-AAAAGCAACACAAATAA (CA)A-3) in 25 µL, respectively, targeting the 12S rDNA gene. The amplification products were visualised in a 2.5% agarose gel, previously stained with ethidium bromide. 

Statistical analysis was performed using Microsoft Excel GraphPad Software and Fischer’s exact test, in order to establish the possible statistically significant differences among the recorded epidemiological data. The difference was considered significant when the *p*-value was ≤0.05.

## 5. Conclusions

Canine dirofilariosis is continuously expanding in Romania, and the positivity rates for circulating microfilariae of *D. immitis* and *D. repens* are very similar—11.95% and 11.58% respectively.

The results of the present investigation suggest possible zoonotic risks for humans living in the screened area. It has consequently become imperative to increase awareness among both veterinarians and physicians regarding the continuous spread of these zoonotic filariae.

## Figures and Tables

**Table 1:** Number and prevalence of canine *Dirofilaria* infections and co-infections in the studied counties from western and south-western Romania.

CRT. NO.	COUNTIES	NO. SAMPLES	NO. POSITIVE	PREVALENCE (%)	*D. immitis* (NO.)	*D. repens* (NO.)	*A. reconditum* (NO.)	*D. immitis +* *D. repens* (NO.)
1	Arad	47	12	25.53	3	9	0	0
2	Bihor	33	2	6.06	0	2	0	0
3	Caras Severin	79	5	6.33	1	4	0	0
4	Dolj	75	3	4.00	1	2	0	0
5	Gorj	62	2	3.23	0	2	0	0
6	Hunedoara	100	4	4.00	1	3	0	0
7	Mehedinti	42	7	16.67	3	4	0	0
8	Olt	52	5	9.62	1	4	0	0
9	Timis	537	186	34.64	93	69	1	23
10	Valcea	60	6	10.00	3	3	0	0
11	Bucuresti	1	1	-	0	0	0	1
TOTAL		1088	233	21.42	106	102	1	24

Legend: no-number.

**Table 2:** Distribution of the dog population according to the studied risk factors.

Epidemiological Data	Positive		Negative
	NO.	%	NO.
Age			
≤1 years (*n* = 123)	13	10.57	110
1 to ≤3 years (*n* = 238)	54	22.69	184
3 to ≤6 years (*n* = 214)	71	33.18	143
>6 years (*n*= 202)	65	32.18	137
NA * (*n* = 311)	30	9.65	281
Gender			
Female (*n* = 371)	82	22.10	289
Male (*n* = 436)	151	34.63	285
NA (*n*= 281)	0	0.00	281
Breed			
Purebreed (*n* = 276)	88	31.88	188
Crossbreed (*n* = 531)	145	27.31	386
NA (*n* = 281)	0	0.00	281
Habitat			
Urban (*n* = 727)	126	17.33	601
Rural (*n* = 361)	107	29.64	254
Owner/shelter			
owner (*n* = 1028)	173	16.83	855
shelter (*n* = 60)	60	100.00	0
Total	233	21.42	855
	1088		

* NA—not available.

**Table 3:** The statistical analysis between positive and negative cases according to the studied risk factors.

Epidemiological Risk Factors	Compared Groups	*p*-Value	*p*-Value Interpretation
Age	≤1 year vs. 1 to ≤3 years	0.0044	very statistically significant
	≤1 year vs. 3 to ≤6 years	0.0001	highly statistically significant
	≤1 year vs. >6 years	0.0001	highly statistically significant
	1 to ≤3 years vs. 3 to ≤6 years	0.0153	statistically significant
	1 to ≤3 years vs. >6 years	0.0311	statistically significant
Gender	female vs. male	0.0001	highly statistically significant
Breed	purebred vs. crossbreed	0.1903	not statistically significant
Habitat	urban vs. rural	0.0001	highly statistically significant
Owner/shelter	owner vs. shelter	0.0001	highly statistically significant
